# *Edgeworthia gardneri* (Wall.) Meisn. Water Extract Ameliorates Palmitate Induced Insulin Resistance by Regulating IRS1/GSK3β/FoxO1 Signaling Pathway in Human HepG2 Hepatocytes

**DOI:** 10.3389/fphar.2019.01666

**Published:** 2020-01-30

**Authors:** Yi Zhang, Li Shan Yan, Yu Ding, Brian Chi Yan Cheng, Gan Luo, Jing Kong, Tong Hua Liu, Shuo Feng Zhang

**Affiliations:** ^1^ School of Chinese Materia Medica, Beijing University of Chinese Medicine, Beijing, China; ^2^ College of Professional and Continuing Education, Hong Kong Polytechnic University, Hong Kong, China; ^3^ Chinese Medicine Department of Quality Healthcare Medical Services , Hong Kong, China; ^4^ Tibetan Medicine Department of Tibetan Traditional Medical College, Lhasa, China

**Keywords:** *Edgeworthia gardneri*, insulin resistance, palmitate, IRS1/GSK3β/FoxO1 signaling pathway, HepG2 cells

## Abstract

The flower of *Edgeworthia gardneri* (Wall.) Meisn is commonly used in beverage products in Tibet and has potential health benefits for *diabetes*. However, the mechanisms underlying anti-insulin resistance (IR) action of the flower of *E. gardneri* are not fully understood. This study aims to investigate the effects of the water extract of the flower of *E. gardneri* (WEE) on IR in palmitate (PA)-exposed HepG2 hepatocytes. WEE was characterized by UPLC analysis. PA-treated HepG2 cells were selected as the IR cell model. The cell viability was determined using MTT assay. Moreover, the glucose consumption and production were measured by glucose *oxidase* method. The glucose uptake and glycogen content were determined by the 2-NBDG (2-deoxy-2-[(7-nitro-2,1,3-benzoxadiazol-4-yl) amino]-D-glucose) glucose uptake assay and anthrone-sulfuric acid assay, respectively. The intracellular triglyceride content was detected by oxidative enzymic method. Protein levels were examined by Western blotting. Nuclear localization of FoxO1 was detected using immunofluorescence analyses and Western blotting. The expression of FoxO1 target genes was detected by quantitative real-time polymerase chain reaction (qRT-PCR). The viability of PA-treated HepG2 cells was concentration-dependently increased by incubation with WEE for 24 h. WEE treatment remarkably increased the consumption and uptake of glucose in PA-exposed HepG2 cells. Moreover, treatment with WEE significantly decreased the PA-induced over-production of glucose in HepG2 cells. After exposure of HepG2 cells with PA and WEE, the glycogen content was significantly elevated. The phosphorylation and total levels of IRβ, IRS1, and Akt were upregulated by WEE treatment in PA-exposed HepG2 cells. The phosphorylation of GSK3β was elevated after WEE treatment in PA-treated cells. WEE treatment also concentration-dependently downregulated the phosphorylated CREB, ERK, c-Jun, p38 and JNK in PA-exposed HepG2 cells. Furthermore, the nuclear protein level and nuclear translocation of FoxO1 were also suppressed by WEE. Additionally, PA-induced changes of FoxO1 targeted genes were also attenuated by WEE treatment. The GLUT2 and GLUT4 translocation were also promoted by WEE treatment in PA-treated HepG2 cells. Taken together, WEE has potential anti-IR effect in PA-exposed HepG2 cells; the underlying mechanism of this action may be associated with the regulation of IRS1/GSK3β/FoxO1 signaling pathway. This study provides a pharmacological basis for the application of WEE in the treatment of metabolic diseases such as type 2 diabetes mellitus.

## Introduction

Type 2 diabetes mellitus (T2DM) is one of the most prevalent metabolic diseases worldwide and characterized by hyperglycemia accompanied by progressive and irreversible loss of pancreatic insulin secretion (Ashcroft and Rorsman, 2012). Insulin resistance (IR), a subnormal biological response to normal insulin concentrations in target cells (e.g., hepatocytes, skeletal muscle cells, and adipocytes), plays a pivotal role in the development of several metabolic diseases including T2DM, hypertension, and hyperlipidemia (Reaven, 1991; Lillioja et al., 1993; Veerkamp et al., 2005). IR is also regarded as the risk factor of different diseases, such as nephropathy, retinopathy, myocardial infarction, and neuropathy (Kahl and Roden, 2018). In view of the adverse health consequences associated with IR, the search for safe and effective anti-IR drugs has been an area of intensive research.

The pathogenic mechanism underlying IR is complex and closely related to increased amounts of free fatty acids (FFAs) in plasma (Boden et al., 2001). In patients with T2DM, circulating FFAs directly enter into the liver; increased levels of hepatic FFAs can thus induce IR in the liver and activate some serine kinases, such as c-Jun-N-terminal kinase, p38, and ERK (Bi et al., 2013; Gao et al., 2018), leading to dephosphorylation of insulin receptor and its substrate 1/2 (IRS-1/2), thereby inhibiting Akt activity (Nakamura et al., 2009a). Subsequently, the downstream protein GSK3β was activated and the phosphorylation of FoxO1 was inhibited, resulting in the translocation of FoxO1 into the nucleus and triggers the hepatic IR (Cao et al., 2014). Therefore, targeting the IRS1/GSK3β/FoxO1 signaling is one of the focuses in developing effective anti-IR agents for T2DM.

Natural supplements probably represent an ideal source to explore safe and effective agents for the treatment of IR (Kouzi, 2015; Li et al., 2019). The flower of *Edgeworthia gardneri* Wall. Meisn (Lv Luo Hua in Chinese) has been used to prepare an herbal tea that is commonly consumed as a healthy beverage in Tibet to ameliorate the metabolic disorders (Xu et al., 2012; Zhang et al., 2019b). It has reported that the extracts of the flower of *E. gardneri* has a wide array of pharmacological activities, such as anti-hyperglycemia, anti-adipogenesis, and α-glucosidase inhibition (Ma et al., 2015; Gao et al., 2016; Zhang et al., 2019b). However, scant reports have been issued on the anti-IR property of the water extract of flower of *E. gardneri* (WEE) and the underlying mechanism of this action remains unclear. To provide pharmacological justiﬁcation for the use of WEE as an anti-IR agent, we employed a classic IR cell model called the palmitate (PA) induced HepG2 hepatocytes to investigate the anti-IR effects of WEE and to explore the underlying mechanisms.

## Materials

3-[4,5-Dimethylthiazol-2-yl]-2,5 diphenyl tetrazolium bromide (MTT) and sodium salt of PA were bought from Sigma Chemical Co (St. Louis MO, USA). Fatty acid free bovine serum albumin was obtained from *Yeasen* Biotech Co., Ltd (Shanghai, China). Penicillin-streptomycin solution was obtained from Caisson labs (Smithfield, UT, USA). Dulbecco’s Modified Eagle Medium (DMEM) was acquired from Corning Cellgro (Manassas, VA, USA). Fetal bovine serum (FBS) was purchased from Biological Industries Co. (Beth-Haemek, Israel). 2-Deoxy-2-[(7-nitro-2,1,3-benzoxadiazol-4-yl) amino]-D-glucose (2-NBDG) was purchased from Thermo Fisher Scientific Inc. (Waltham, MA, USA). Phospho-IRS1 (Tyr 612, catalog no. PAB12628) was purchased from Abnova (Taiwan, China). Phospho-IGF-I receptor β/insulin receptor β (Tyr1131/Tyr1146, catalog no. 3021), insulin receptor β (4B8, catalog no. 3025), phospho-FoxO1 (Ser256, catalog no. 9461), FoxO1 (C29H4, catalog no. 2880), GSK-3β, p-Akt (ser473, catalog no. 4060), Akt (catalog no. 9272), c-Jun (catalog no. 9165), phospho-c-Jun (Ser73, catalog no. 9164), extracellular signal-regulated kinase (ERK catalog no. 4695), phospho-ERK (Thr202/Tyr204, catalog no. 4370), c-Jun N-terminal kinase (JNK, catalog no. 9252), phospho-JNK (Thr183/Tyr185, catalog no. 4668), phospho-p38 (Thr180/Tyr182), p38 mitogen-activated protein kinase (p38, catalog no. 8690), phospho-CREB (Ser133, catalog no. 9198), CREB (catalog no. 9197), GLUT4 (catalog no. 2213), Na, K-ATPase (catalog no. 3010), β-Actin (catalog no. 4967) and anti-rabbit IgG HRP linked anti-body (catalog no. 7074), and alexa fluor 488-conjugated secondary antibody (catalog no. 4412) were bought from Cell signaling technology (Boston, MA, USA). sp1 monoclonal antibody (catalog no. sc-420) and anti-mouse IgG HRP-linked antibody (catalog no. sc-516102) were purchased from Santa Cruz Biotechnology (Santa Cruz, CA, USA). GLUT2 (catalog no. ab54460) and phospho-GSK-3β (Ser9, catalog no. 131097) were bought from Abcam (Cambridge, UK). Rutin, chlorogenic acid and metformin were purchased from *Shanghai Yuanye* Biological Technology Co. Ltd. (Nanjing, China). Tiliroside was obtained from National Institutes for Food and Drug Control (Beijing, China). The purity of each standard was higher than 98%.

## Methods

### Preparation of WEE Powder

The flower of *E. gardneri* was purchased from Sichuan Hao rui jia Biotechnology Co., Ltd (Chengdu, China) and authenticated by Lecturer Guang Xi Ren (School of Chinese Materia Medica, Beijing University of Chinese Medicine). Voucher specimens have been deposited at the Department of Pharmacology, School of Chinese Medicine, Beijing University of Chinese Medicine. The flower of *E. gardneri* was grounded and macerated for 1 h with 500 ml of water at room temperature, and then reﬂuxed three times for 1 h each time. The combined extracts were filtered and concentrated by rotary evaporation under reduced pressure to remove the solvent. The concentrated extracts were rapidly frozen at −80°C, and then dried in a freeze-dryer (FD-1D-80, Biocool, Beijing, China). The yield of powdered WEE was 19.8%. The samples after ultra-performance liquid chromatography (*UPLC*) analysis were diluted by 50% methanol-water solution and methanol, respectively. Then the diluent was filtered through a Millipore membrane filter with an average pore diameter of 0.45 μm.

### Characterization of WEE Powder

To control the quality of the WEE, *UPLC* analysis was conducted using a Waters Acquity UPLC system (*Waters* Corporation, Milford, MA, USA), consisting of a binary solvent delivery pump, an auto sampler, and a photodiode array detector. The chromatographic separation was performed using a Waters Acquity BEH 100 × 2.1 mm, 1.7 μm, C18 column with a gradient mobile phase of solvent A (acetonitrile) and solvent B (0.2% phosphoric acid water). The UPLC elution profile was as follows: 0–5 min, 8%A; 5–7 min, 8%–13%A; 7–15 min, 13–16%A; 15–17 min, 16%–22%A; 17–22 min, 22%–25%A; 22–25 min, 25%–40%A; 25–26 min, 40%A. The flow rate was maintained at 0.3 ml·min^-1^ and the column temperature was set at 30°C. The chromatograms were monitored with the PDA detector at a wavelength of 340 nm to detect rutin, chlorogenic acid, and tiliroside. Each sample of 5 μl was injected for analysis.

### Cell Culture and PA Treatment

The HepG2 cell line was obtained from American Type Culture Collection (Manassas, VA, USA). Cells were incubated in the DMEM medium at 37°C with 5% CO_2_. Penicillin/streptomycin antibiotics (1%) and heat-inactivated FBS (10%) were added into the DMEM medium. To induce IR, 0.3 mM PA (sodium salt of PA, Sigma, Malaysia) was added to HepG2 cells with fatty acid free BSA as supplement in a final concentration of 1% in the culture medium as previously described (Ma et al., 2017). After reaching 70–80% confluence, the cells were washed with PBS twice and incubated with normal glucose (5.5 mM) or high glucose (30 mM) plus PA (0.3 mM) in the absence or presence of WEE or Metformin with various concentrations for 24 h. Cells treated with normal glucose (5.5 mM) were used as the negative control and cells treated with metformin were used as the positive control. To prepare the sample solution for bioassays, WEE and metformin were freshly dissolved in DMEM, ﬁltered through a 0.22 μm syringe ﬁlter, and then diluted with cell culture medium to various concentrations.

### Cell Viability Assay

The effect of WEE on PA-induced HepG2 cells viability was measured by MTT assay. Briefly, 8 × 10^3^ cells were seeded in 96 well plates and allowed to adhere overnight. After the treatments for 24 h followed by insulin (100 nM) incubation for 20 min, 10 μl of MTT solution (5 mg/ml) was added in each well and incubated for 3 h at 37°C. The supernatant was then removed and the remaining formazan crystals were dissolved with 100 μl of DMSO in each well. The absorbance at 570 nm was determined using a microplate spectrophotometer (BMG SPECTROstar Nano, Germany). The value of cell viability in the control group was set as 100%. Six replicate wells were used.

### Cell Counting

HepG2 cells were seeded in 6 wells (1.5 × 10^5^ cells) for 24 h. After the treatments for 24 h followed by insulin (100 nM) incubation for 20 min, the number of cells in each well were calculated by a hemocytometer. There were six replicates for each treatment.

### Glucose Uptake Assay

The glucose uptake was determined as previously reported with some modiﬁcation (Nie et al., 2017). Briefly, HepG2 cells (4 × 10^4^ cells/well) were cultured in a chamber slide (Thermo Scientific, Waltham, MA, USA). The 24 h treatments were followed by insulin (100 nM) for 30 min and 0.3 mM 2-NBDG for 45 min incubation at 37°C. The cells were then washed three times by PBS and then incubated with Hoechst 33342 (Solarbio, Beijing, China) for 30 min. After being washed with PBS three times, the cells were incubated with Hank’s solution. Images were obtained using identical acquisition settings on a Nikon A1R Eclipse Ti confocal microscope (Nikon Corp., Tokyo, Japan). The fluorescence intensity and area were analyzed using Image J software (National Institutes of Health [NIH], Bethesda, MD, USA) as previously described (Bankhead, 2014). The fluorescence intensity and area were normalized with the cell number. Seven images for each group were calculated.

### Glucose Consumption Assay

Glucose consumption was determined according to previous study with some modiﬁcation (Yan et al., 2016). Brieﬂy, HepG2 cells (6 × 10^3^ cells/well) were cultured in a 96-well plate with six wells for RPMI-1640 medium left as blanks. Treatments for 24 h were followed by insulin (100 nM) for 30 min. The cells were washed with PBS twice and the medium was replaced by RPMI-1640 containing 11.1 mmol/L glucose supplemented with 0.2% BSA. After 24 h incubation, a quantity of 10 μl of medium was sampled and glucose consumption was calculated by the glucose concentration of blank wells minus glucose concentrations in plated wells by using the glucose kit (Nanjing Jian cheng, Nanjing, China) according to the instruction. The MTT assay was used to adjust the glucose consumption. There were six replicates for each treatment.

### Triglyceride Content Assay

HepG2 cells were seeded in 6 wells (1.5 × 10^5^ cells) for 24 h. After 24 h treatments, cells were washed by PBS and then lysed with cold Radio Immunoprecipitation Assay (RIPA) protein extraction buffer containing 1% protease and phosphatase inhibitors (Beyotime biotechnology, Beijing, China) for 30 min on ice. The content of triglyceride (TG) was determined by TG assay kit (BioAssay systems, Hayward, CA, USA) according to the manufacturer’s instruction. TG concentration was normalized with the total protein content determined from the whole cell lysates. There were six replicates for each treatment.

### Glycogen Content Assay

The glycogen content assay was conducted as previously described with some modiﬁcation (Nie et al., 2017). HepG2 cells were seeded in 60 mm culture dishes (8 × 10^5^ cells) for 24 h. After 24 h treatments, cells were washed by PBS and incubated insulin (100 nM) for 20 min. Then glycogen content of cells was estimated by glycogen assay kit (Solarbio, Beijing, China) using the sulfuric acid-anthrone colorimetric method following the manufacturer’s approach. The glycogen content was normalized with the cell amount. Six replicates were used.

### Determination of Glucose Production

Glucose production assay was performed according to the published procedure (Ishii et al., 2015). Briefly, cells were seeded in 24 well plates and incubated for 24 h. The 24 h treatments were followed by insulin (100 nM) for 20 min; the cells were washed three times with PBS to remove glucose. The cells were then incubated with 1 nM insulin for 16 h in 300 μl of glucose production medium (glucose- and phenol red-free DMEM containing gluconeogenic substrates, including 20 mM sodium lactate and 2 mM sodium pyruvate). A quantity of 10 μl of medium was sampled for measurement of glucose concentration using a glucose assay kit (*BioAssay Systems*, Hayward, CA, USA). Glucose concentration was normalized with the total protein content determined from the whole cell lysates.

### Cytoplasmic and Nuclear Proteins Extraction

Cytoplasmic and nuclear proteins were extracted using nuclear extraction kit (Solarbio, Beijing, China) according to the manufacturer’s instructions. Briefly, HepG2 cells were seeded in 100 mm diameter culture dishes (1 × 10^6^ cells) and incubated for 24 h. After 24 h treatments followed by insulin (100 nM) incubation for 20 min, cells were harvest and washed with PBS. Ice-cold cytoplasmic protein extraction solution was added to separate cytoplasmic and nuclear protein after centrifugation. Ice-cold nuclear protein extraction solution was then added to nuclear fraction for protein extraction. Three replicates were used.

### Plasma Membrane Protein Extraction

Plasma membrane protein was extracted using a plasma membrane protein isolation kit (Invent Biotechnologies, Inc., Eden Prairie, MN, USA). Briefly, HepG2 cells were seeded in 100 mm diameter culture dishes (1 × 10^6^ cells) and incubated for 24 h. After 24 h treatments, the cells were incubated in the absence or presence of insulin (100 nM) for 20 min. Cells were harvested and washed with PBS. The plasma membrane fraction was separated from the cellular components (nuclei, cytosol, and organelles) according to the manufacturer’s instructions. Three replicates were used.

### Western Blot Analysis

Western blot was performed as previously described (Zhang et al., 2019a). Briefly, HepG2 cells were seeded in 60 mm culture dishes (5 × 10^5^ cells) for 24 h. After the treatments for 24 h followed by insulin (100 nM) incubation for 20 min, cells were washed with cold PBS, and then lysed with cold Radio Immunoprecipitation Assay (RIPA) protein extraction buffer containing 1% protease and phosphatase inhibitors (Beyotime biotechnology, Beijing, China) for 30 min on ice. An aliquot of 20 μg of the supernatant protein from each sample was heated with 4 × sodium dodecyl sulfate (SDS) sample buffer at 95 °C for 8 min, and separated electrophoretically on a 10% SDS–polyacrylamide gel. Subsequently, proteins were transferred onto PMSF membranes for 3 h and blocked for 1 h. PMSF membranes were then exposed to indicate primary antibodies in blocking buffer at 1:1,000 dilutions overnight at 4°C. The membranes were then incubated with the anti-rabbit IgG secondary antibody or and anti-mouse IgG HRP-linked antibody at 1:2,000 dilutions for 1 h. Visualization was performed using Tanon 5200 Multi chemiluminescent imaging system (Tanon Science & Technology Co., Ltd., Shanghai, China) with enhanced-chemiluminescence substrate, and the blots were analyzed using Image J software. Protein levels were corrected with values determined on β-actin blots. Three replicates were used.

### Immunofluorescence Staining

Immunofluorescence staining was conducted to detect FoxO1 localization after WEE treatment. Briefly, 5 × 10^4^ cells were cultured on chamber slide overnight. The 24 h treatments were followed by insulin (100 nM) stimulation for 20 min; the cells were fixed with formaldehyde in PBS (w:v, 4%) for 15 min and washed with PBS and permeabilized with Triton X-100 (0.25%) during 30 min at 37 °C. Cells were then blocked for 1 h with BSA (2%) and incubated with FoxO1 (1:800) overnight at 4°C, respectively. After washing with cold PBS, cells were incubated with to Alexa Fluor 488-conjugated secondary antibody (1:500) for 1 h at room temperature. Coverslips were mounted with DAPI (YEASEN, Shanghai, China) and visualized in Nikon A1R Eclipse Ti confocal microscope (Nikon Corp., Tokyo, Japan).

### RNA Extraction and Quantitative Real-Time Polymerase Chain Reaction (qRT-PCR)

RT-PCR was conducted as previously reported (Luo et al., 2019). Briefly, HepG2 cells were seeded in 6 well plates (4 × 10^5^ cells) for 24 h. Treatments for 24 h were followed by insulin (100 nM) incubation for 20 min. Total ribonucleic acid (RNA) was extracted from the HepG2 cells using TRIzol reagent (Thermo Fisher Scientific, Waltham, MA, USA) with Trizol reagent (Invitrogen, USA) and reverse-transcribed into cDNA using Prime Script TM RT reagent Kit (Takara, Japan) according to the manufacturer’s protocol. Quantitative RT-PCR was performed in triplicate using PowerUp™ SYBR™ Green master mix (Thermo Fisher Scientific, Waltham, MA, USA) with a Step One Plus Real Time PCR system (Applied Biosystems, CA, USA). **Table 1** shows the primer sequences of various genes (Pdk4, Dhrs9, Rbp1, and GAPDH). The data were analyzed by relative quantitation using the ΔΔCt method and normalized to the endogenous control GAPDH.

**Table 1:** Primer sequences for real-time PCR.

Gene	Forward Primer (5’-3’)	Reverse Primer (5’-3’)
Pdk4	GGAGCATTTCTCGCGCTACA	ACAGGCAATTCTTGTCGCAAA
Dhrs9	CTGTGGACTCGTAAAGGAAAACT	GCAGCGATTACATGAAATCCCT
Rbp1	TCCAGTCACTCCCCGAAATG	AGGTACTCCTCGAAATTCTCGTT
GAPDH	GGTGTGAACCATGAGAAGTATGA	GAGTCCTTCCACGATACCAAAG

### Statistical Analysis

Statistical analysis was performed using GraphPad Prism version 5.0 (GraphPad software, San Diego, CA, United States). The data are presented as mean ± standard error of mean (SEM). Statistical significance was determined by one-way ANOVA followed by the Dunnett’s multiple comparisons. *p* < 0.05 was considered as statistically signiﬁcant.

## Results

### Characterization of WEE Powder

**Figure 1:**
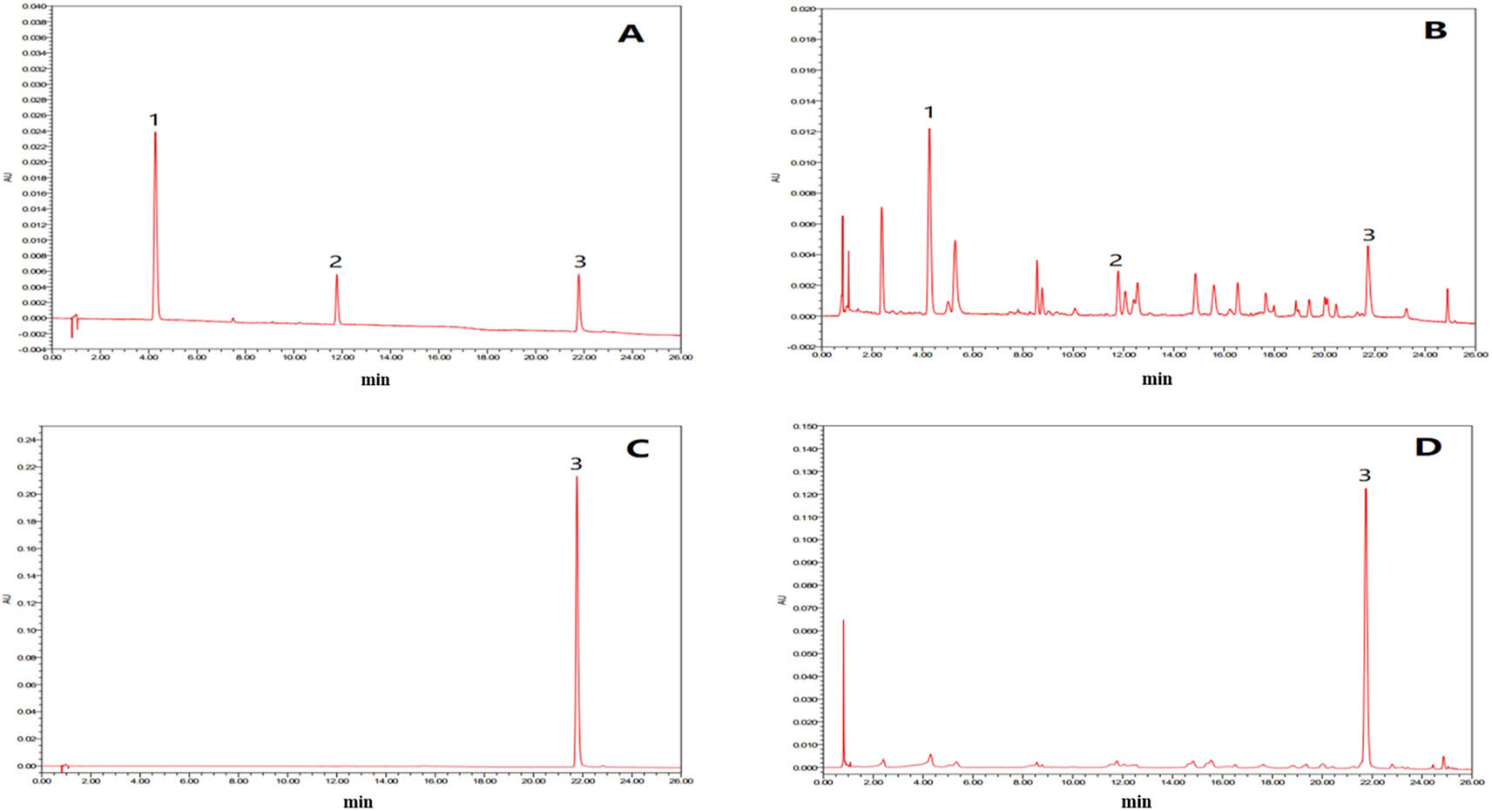
UPLC chromatograms of chlorogenic acid, rutin, and tiliroside in WEE. **(A)** UPLC chromatogram of rutin, chlorogenic acid, and tiliroside dissolved in 50% methanol. **(B)** UPLC chromatogram of WEE sample dissolved in 50% methanol. **(C)** UPLC chromatogram of WEE sample dissolved in methanol. **(D)** HPLC chromatogram of WEE sample dissolved in methanol. Peaks 1, 2, and 3 represent chlorogenic acid, rutin, and tiliroside, respectively.

In this study, the UPLC chromatogram showed that rutin, chlorogenic acid, and tiliroside were present in WEE powder (see **Figures 1A–D**). The mean contents of rutin, chlorogenic acid, and tiliroside occurring in WEE powder were 1.6, 5.1, and 32.5 mg/g, respectively (see **Table 2**).

**Table 2:** The contents of chlorogenic acid, rutin, and tiliroside in WEE.

Analytes	Regression equation	Correlation (r)	Contents in sample (mg/g)
Chlorogenic acid	Y=5949015.99X+581.86	0.9994	5.1
Rutin	Y=4158022.02X+153.89	0.9992	1.6
Tiliroside	Y=5731563.67X-18725.29	0.9995	32.5

### Effect of WEE on the Viability and the Number of Cells

**Figure 2:**
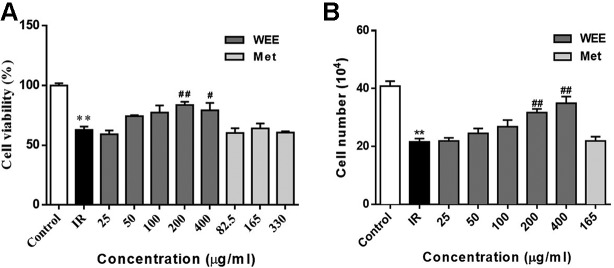
WEE treatment increased the cell viability in PA treated HepG2 cells. HepG2 cells were incubated with normal glucose (5.5 mM) or high glucose (30 mM) with PA (0.3 mM) in the absence or presence of WEE (25–400 μg/ml) or metformin (82.5–330 μg/mL) for 24 h and followed by insulin (100 nM) incubation for 20 min. Cell viability was determined by the MTT assay. Results were expressed as percentages of control **(A)**. The total cell number was calculated by a hemocytometer **(B)**. Data presented in bar charts are mean ± SEM values from six independent experiments. Groups are significantly different from the control group at ^*^ *p* < 0.05, ^**^ *p* < 0.01. The groups are significantly different from the IR group at ^#^ *p* < 0.05 and ^##^ *p* < 0.01 determined by Dunnett’s multiple comparisons test.

The concentrations of WEE used in subsequent experiments were evaluated by cell viability through MTT assays. Compared with the control group, the viability of HepG2 cells was significantly decreased after 24 h treatment with PA. WEE treatment at 25–400 μg/ml obviously increased the viability of HepG2 cells in the presence of PA. Moreover, metformin at concentration up to 330 μg/ml showed no effect on cell viability after being treated with PA (see **Figure 2A**). We also counted the cell number after PA and WEE treatment, and we found that the results of cell number showed the same trend with cell viability (see **Figure 2B**), indicating that WEE treatment increased the total cell number after PA exposure. Therefore, we chose 25, 50, 100, 200, and 300 μg/ml and metformin 165 μg/ml in the following experiments.

### Effect of WEE on the Glucose Uptake and Glucose Consumption

**Figure 3:**
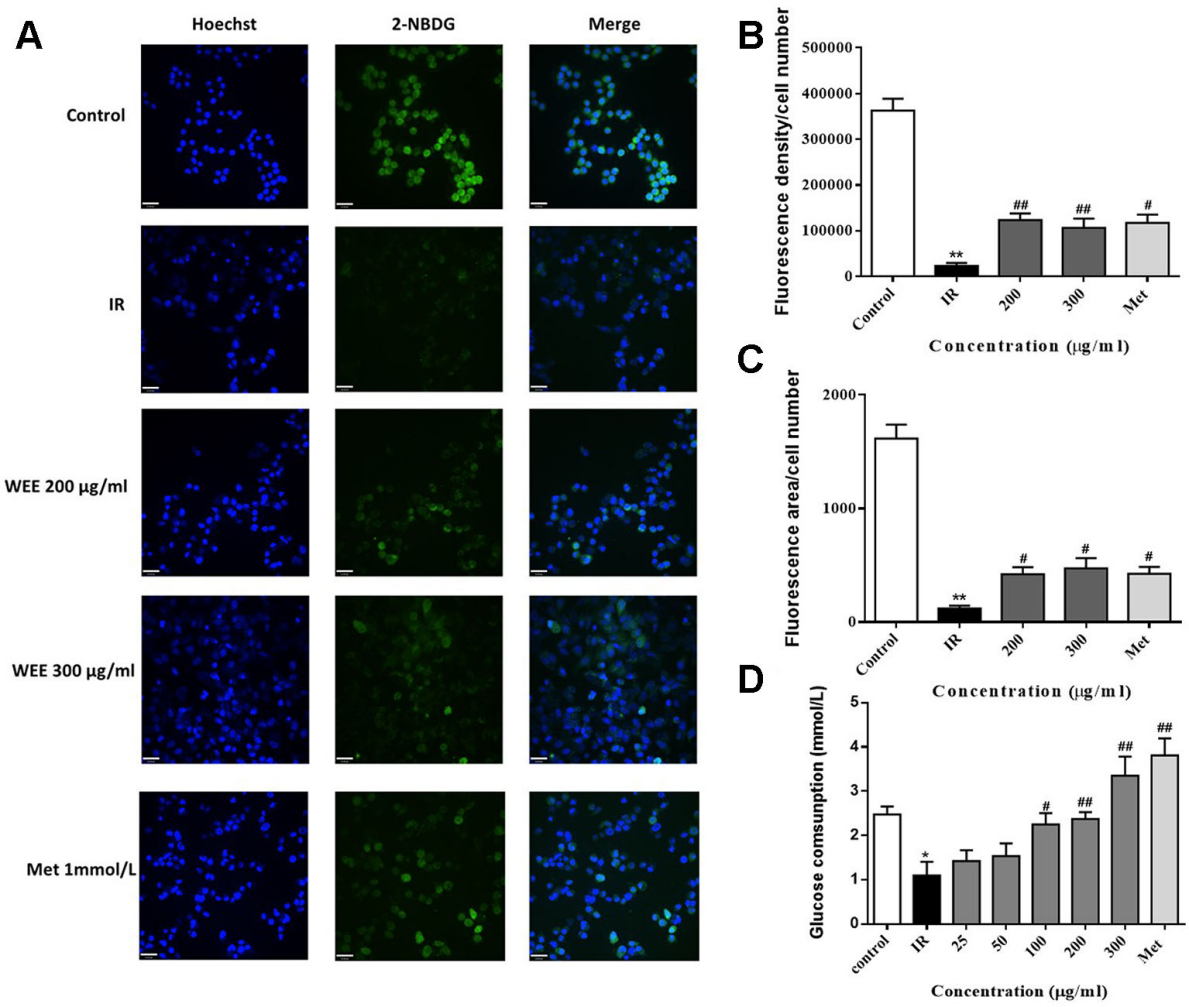
WEE elevated the glucose uptake and consumption in PA-induced HepG2 cells HepG2 cells were incubated with normal glucose (5.5 mM) or high glucose (30 mM) in the absence or presence of WEE (200 and 300 μg/ml) or metformin (165 μg/ml) for 24 h and incubated with insulin (100 nM) for another 30 min. The glucose uptake was then detected by 2-NDBG assay, and the nuclear was stained by Hoechst **(A)**. The fluorescence intensity and area were analyzed using Image J software assays **(B**, **C)**. After 24 h treatment followed by incubation with insulin (100 nM) for 30 min, cells were incubated with RPMI-1640 containing 11.1 mmol/L glucose for 24 h. The glucose contents in the culture medium were determined by glucose assay kit **(D)**. Data presented in bar charts are mean ± SEM values from six independent experiments. Groups are significantly different from the control group at *p < 0.05, **p < 0.01. The groups are significantly different from the IR group at ^#^p < 0.05 and ^##^p < 0.01 determined by Dunnett’s multiple comparisons test. The bar in each photograph indicates 33 μm.

IR is reflected by the rates of reduced glucose uptake into the key insulin-sensitive tissues, such as liver and adipose tissue (Honka et al., 2018). Therefore, glucose uptake and consumption were determined in PA-induced HepG2 cells. Compared with the control group, the glucose uptake was significantly decreased in IR cells, indicated by remarkably reduced fluorescence density and area (see **Figures 3A–C**). WEE treatment concentration-dependently prevented the inhibition of glucose uptake in PA-induced HepG2 cells. Moreover, WEE treatment also obviously increased the glucose consumption in PA exposed HepG2 cells (see **Figure 3D**).

### Effect of WEE on the Glucose Production, the Intracellular Glycogen, and Triglyceride Contents

**Figure 4:**
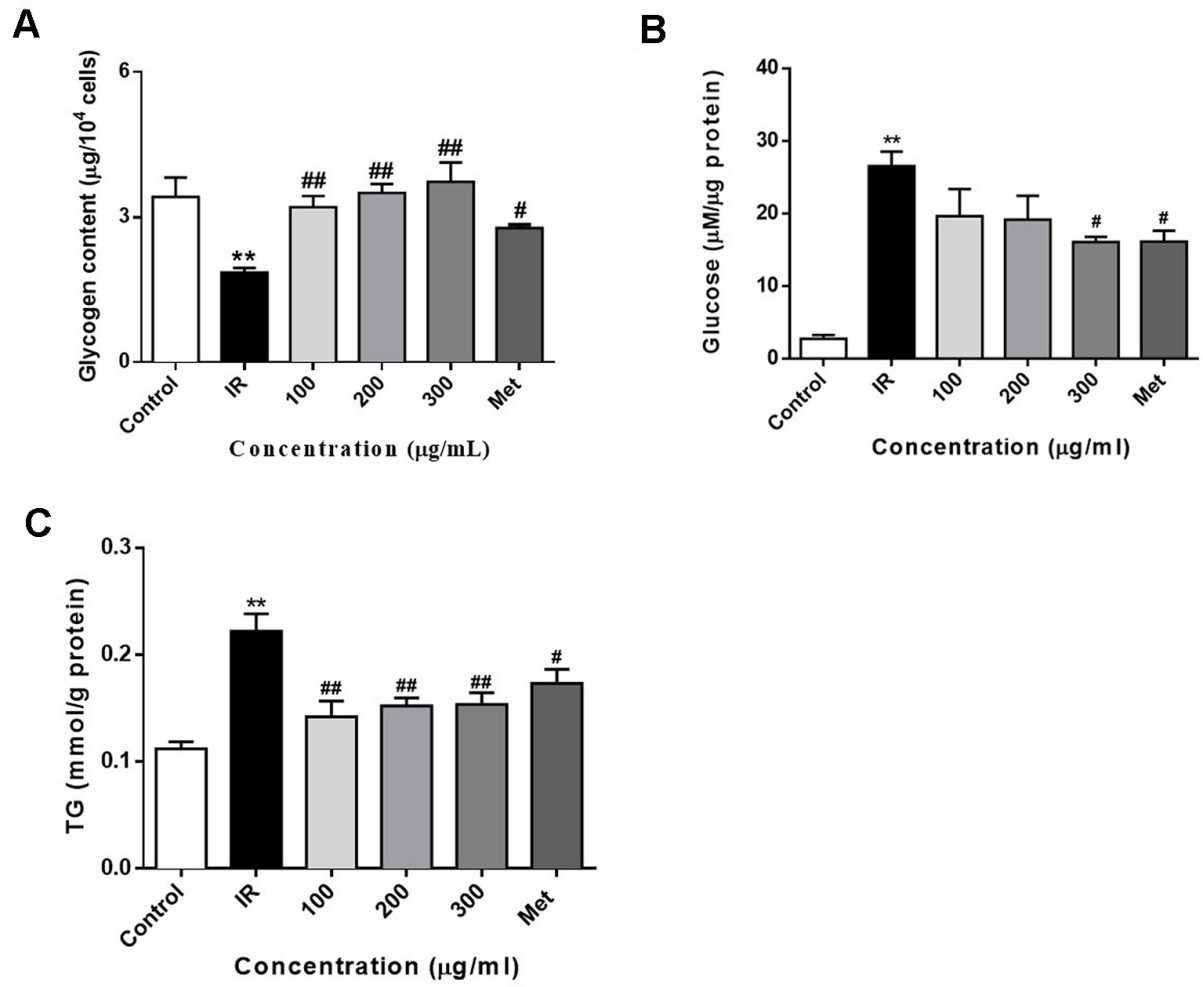
WEE treatment increased the glycogen contents and decreased the glucose production and TG content in PA treated HepG2 cells. HepG2 cells were incubated with normal glucose (5.5 mM) or high glucose (30 mM) plus PA (0.3 mM) in the absence or presence of WEE (100–300 μg/ml) or metformin (165 μg/ml) for 24 h and followed by insulin (100 nM) incubation for 20 min. The intracellular glycogen content was detected by the sulfuric acid-anthrone colorimetric method **(A)**. The glucose production in the culture medium (glucose- and phenol red-free DMEM containing gluconeogenic substrates) was determined by glucose *oxidase* method **(B)**. The intracellular TG content was measured by enzymic method **(C)**. Data presented in bar charts are mean ± SEM values from six independent experiments. Groups are significantly different from the control group at **p < 0.01. The groups are significantly different from the IR group at ^#^p < 0.05 and ^##^p < 0.01 determined by Dunnett’s multiple comparisons test.

Treatment of PA inhibits the glycogen synthesis and activates the gluconeogenesis in HepG2 cells (Ishii et al., 2015; Ma et al., 2017). In this study, we also determined the intracellular glycogen contents and glucose production of HepG2 cells. As shown in **Figure 4A**, exposure of HepG2 cells to 0.3 mM PA for 24 h significantly decreased the glycogen content in HepG2 cells. Moreover, the glycogen content was significantly increased after treatment with PA in combination with WEE. Furthermore, the gluconeogenesis enhanced by PA was concentration-dependently attenuated by the treatment with WEE in HepG2 cells (see **Figure 4B**).

To determine whether WEE decreased lipid accumulation in PA-exposed HepG2 cells, an intracellular TG content assay was performed. Compared with the control group, cells treated with PA for 24 h showed a significant increase in TG content (see **Figure 4C**). WEE treatment markedly suppressed the intracellular TG content in a concentration-dependent manner.

### Effect of WEE on the IRS1/GSK-3β/FoxO1 Signaling Pathway

**Figure 5:**
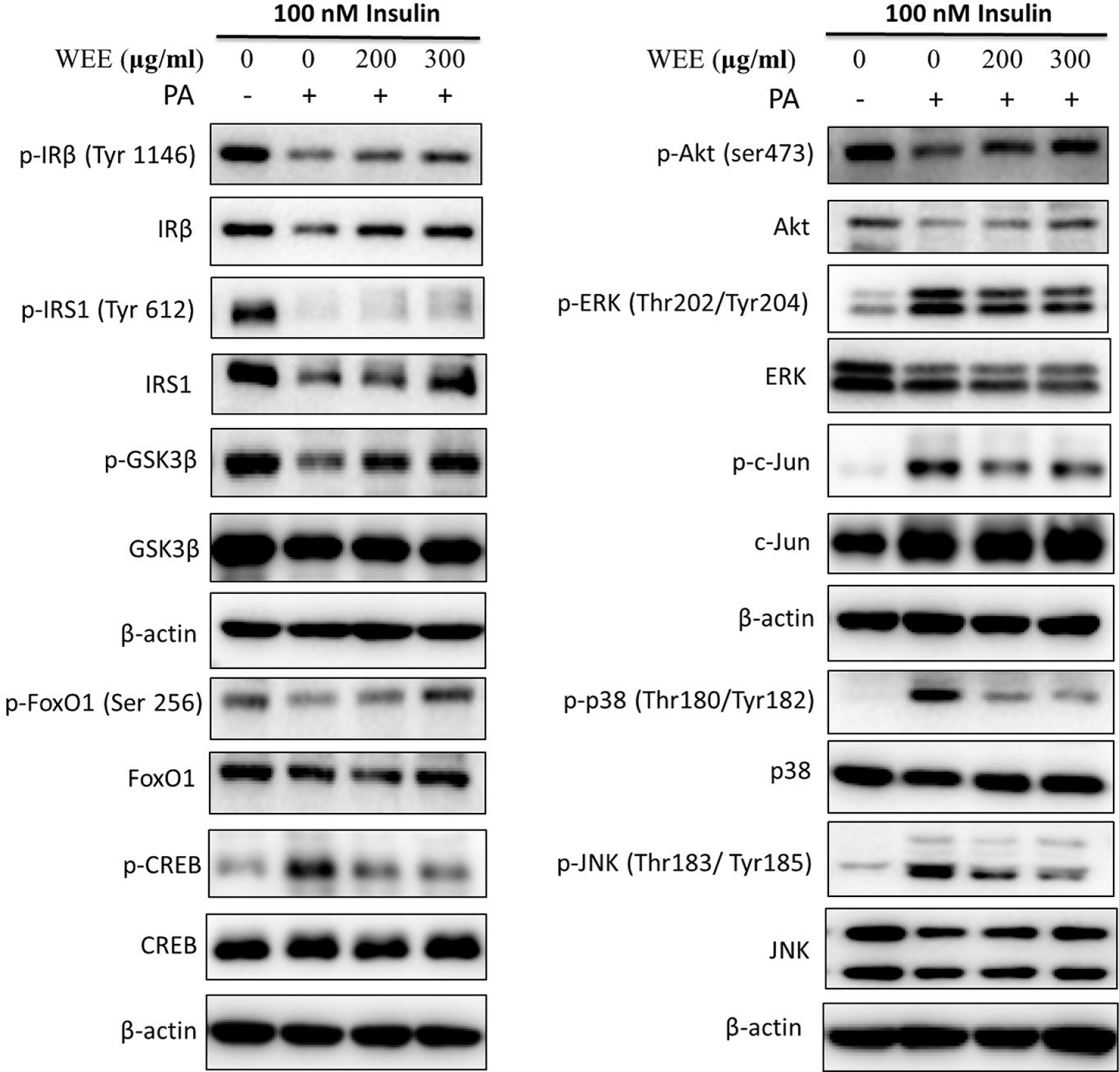
WEE improved the impairment of insulin signaling induced by PA treatment in HepG2 cells. HepG2 cells were incubated with normal glucose (5.5 mM) or high glucose (30 mM) plus PA (0.3 mM) in the absence or presence of WEE (200 and 300 μg/ml) for 24 h and followed by stimulated with insulin (100 nM) for 20 min. Then, the total and phosphorylated forms of IRβ, IRS-1, GSK3β, CREB, c-Jun, FoxO1, Akt, p38, JNK, and ERK were detected by Western blotting.

**Figure 6:**
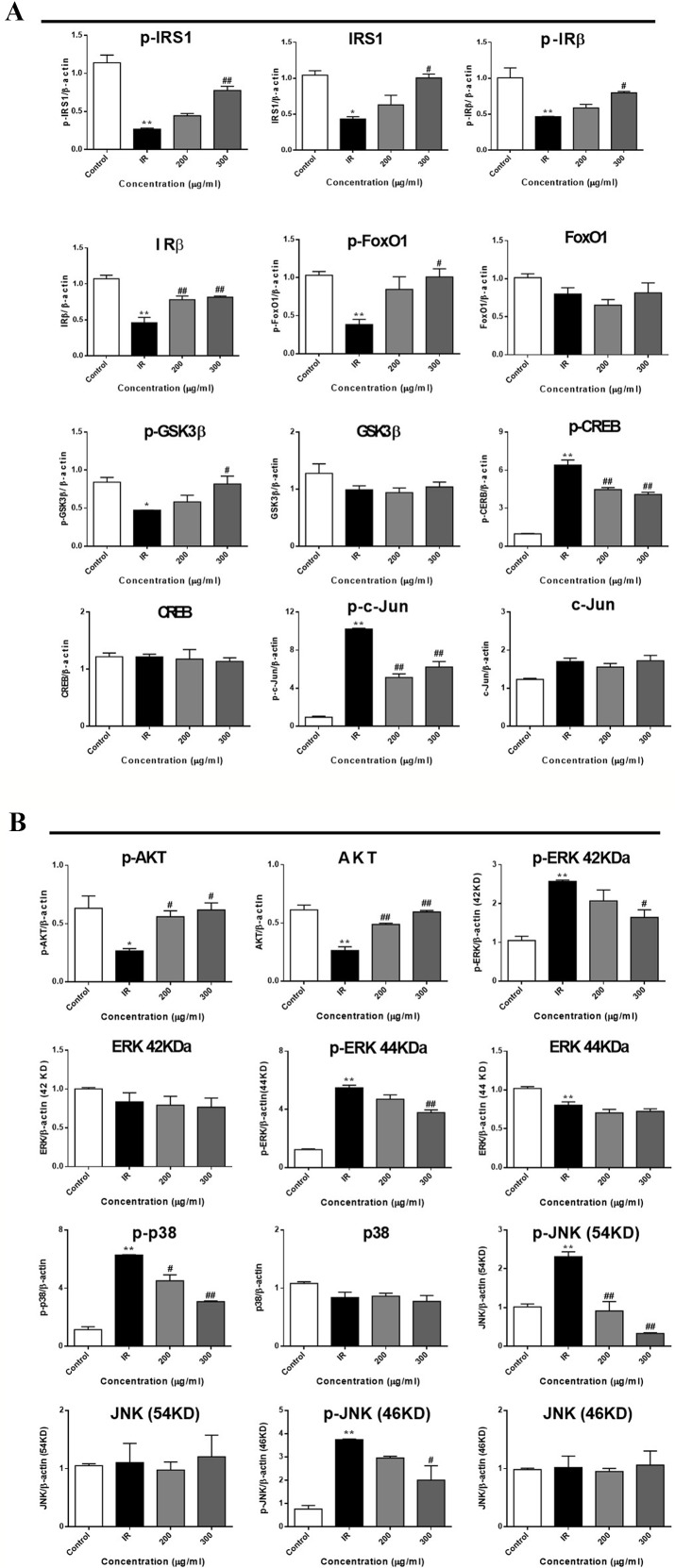
WEE affected the molecular components of IRS1/GSK-3β/FoxO1 pathways in PA treated HepG2 cells. HepG2 cells were incubated with normal glucose (5.5 mM) or high glucose (30 mM) plus PA (0.3 mM) in the absence or presence of WEE (200 and 300 μg/ml) for 24 h and followed by being stimulated with insulin (100 nM) for 20 min. Then, the phosphorylation and total levels of IRβ, IRS-1, GSK3β, CREB, c-Jun, FoxO1, Akt, p38, JNK, and ERK were detected by Western blotting. Bar graphs show the relative expression of indicated proteins. All proteins were normalized for β-actin levels. Data presented in bar charts are mean ± SEM values from three independent experiments. Groups are significantly different from the control group at *p < 0.05, **p < 0.01. The groups are significantly different from the IR group at ^#^p < 0.05 and ^##^p < 0.01 determined by Dunnett’s multiple comparisons test.

To further investigate the mechanism underlying WEE on the PA-induced impairment of insulin signaling molecules, the phosphorylation and expression levels of key molecules in the insulin signaling pathway were determined by immunoblotting. As shown in **Figures 5** and **6**, the phosphorylation of IRβ at Tyr-1146, one of the prominent autophosphorylation sites, was significantly suppressed by PA treatment. Meantime, the downstream molecules of IRβ, such as IRS-1 (Tyr612) and Akt (Ser473), were also decreased after 24 h PA and high glucose treatments in HepG2 cells, indicating that PA is a principal inducer of IR. Moreover, the protein levels of IRβ, IRS-1, and Akt were also marked downregulated by PA and high glucose treatments. The phosphorylation of GSK-3β was also downregulated by PA treatment, and WEE treatment obviously upregulated the phosphorylated GSK-3β. The exposure of HepG2 cells to PA for 24 h significantly elevated the activation of phosphorylated CREB and c-Jun and each phosphorylation level was significantly reduced by WEE treatment. However, the total protein levels of GSK-3β, CREB, and c-Jun were not affected after PA and WEE treatment. We also observed that the phosphorylation of FoxO1, the key transcription factor of insulin signaling pathway, was significantly decreased in IR group, when comparing with the control group. WEE treatment concentration-dependently elevated the phosphorylation of FoxO1. In addition, the phosphorylation of MAPKs, such as ERK, p38, and JNK, were obviously increased in IR cells and these adverse effects were significantly prevented by WEE treatment in a concentration-dependent manner (see **Figures 5** and **6**).

### Effect of WEE on the Nuclear Translocation of FoxO1

**Figure 7:**
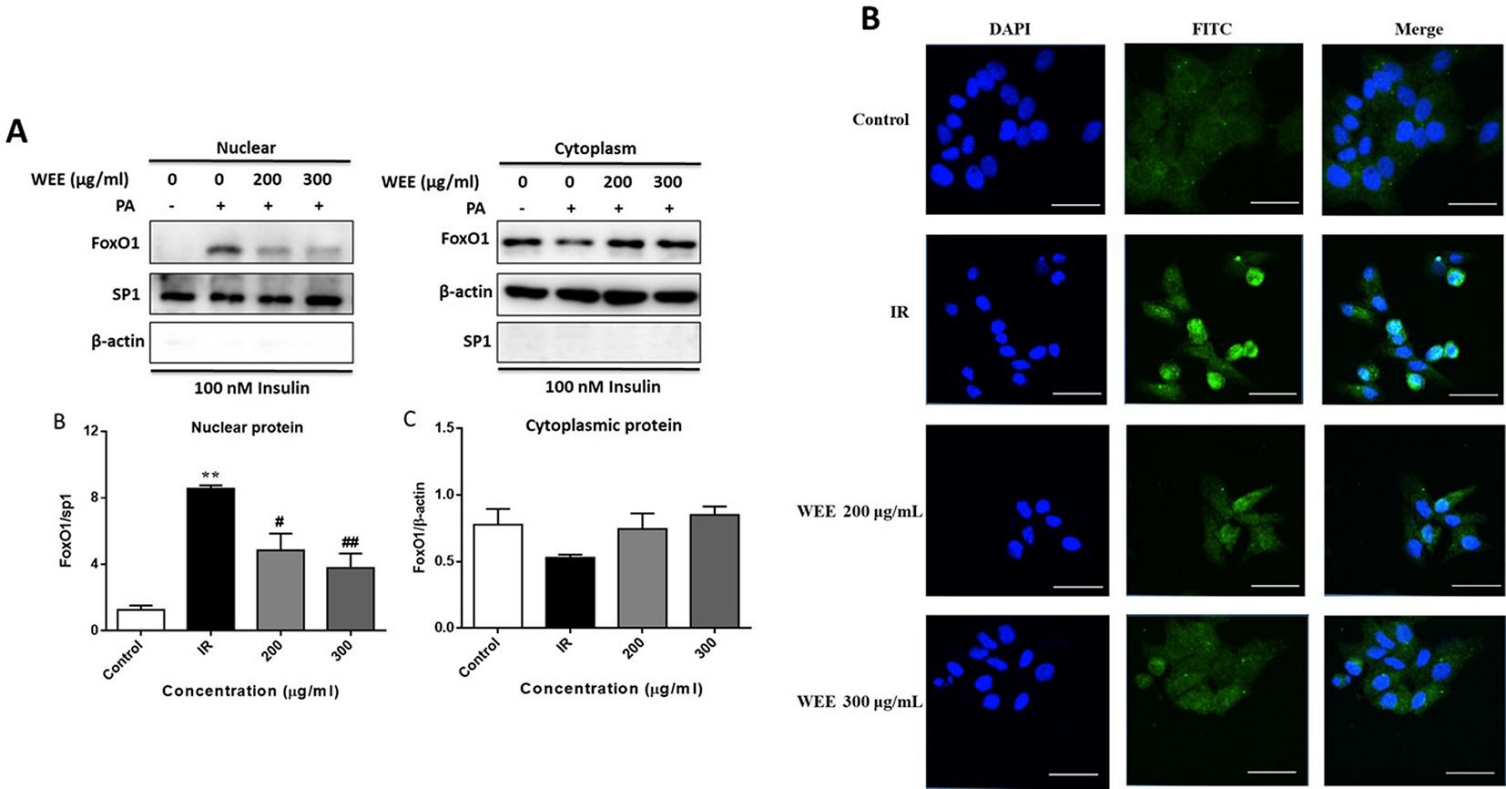
WEE suppressed the nuclear translocation of FoxO1 in PA and high glucose treated HepG2 cells. HepG2 cells were incubated with normal glucose (5.5 mM) or high glucose (30 mM) plus PA (0.3 mM) in the absence or presence of WEE (200 and 300 μg/mL) for 24 h and followed by being stimulated with insulin for 20 min. The cytoplasmic and nuclear protein levels of FoxO1 were determined by Western blotting. Data presented in bar charts are mean ± SEM values from six independent experiments. Groups are significantly different from the control group at **p < 0.01. The groups are significantly different from the IR group at ^#^p < 0.05 and ^##^p < 0.01 determined by Dunnett’s multiple comparisons test **(A)**. The nuclear localization of FoxO1 was determined by immunoﬂuorescence staining. The bar in each photograph indicates 33 μm **(B)**.

Following PA treatment, FoxO1 is translocated into the nucleus and activates its target genes, promoting IR in HepG2 cells (Li et al., 2018). Therefore, we firstly investigated the nuclear translocation of this key transcription factor by Western blot analysis. As shown in **Figure 7A**, the nuclear protein level of FoxO1 was significantly upregulated after PA treatment, and WEE treatment markedly reduced the nuclear accumulation of FoxO1. Comparing with the control group, the cytoplasmic protein of FoxO1 was obviously decreased in IR cells, and WEE treatment concentration-dependently elevated the FoxO1 level in the cytoplasm (see **Figure 7A**). We also found that the sp1 and β-actin expressions in cytosolic and nuclear fractions were very low, respectively, suggesting that pure cytosolic and nuclear fractions were isolated.

Next, we also performed an immunofluorescence assay to examine the nuclear localization of FoxO1. We observed that FoxO1 was normally sequestered in the cytoplasm, and nuclear translocation was nearly not observed in the control cells. However, the nuclear translocation of FoxO1 was significantly induced after 24 h PA treatment, which was remarkably prevented by WEE treatment (see **Figure 7B**).

### Effect of WEE on the FoxO1 Target Genes Expression

**Figure 8:**
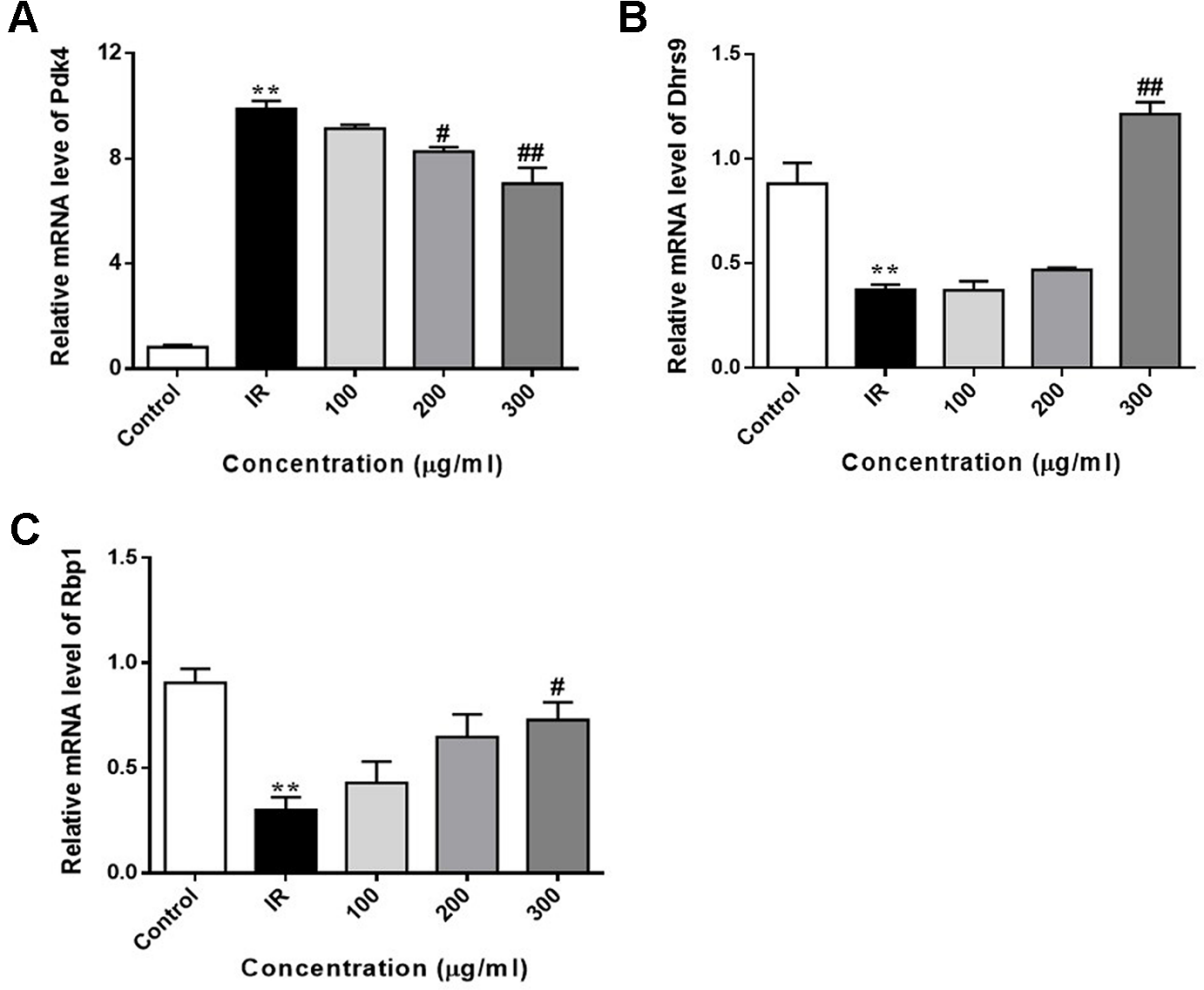
WEE reversed PA induced changes of FoxO1 target genes in PA treated HepG2 cells. HepG2 cells were incubated with normal glucose (5.5 mM) or high glucose (30 mM) plus PA (0.3 mM) in the absence or presence of WEE (200 and 300 μg/ml) for 24 h and followed by being stimulated with insulin (100 nM) for 20 min. The mRNA levels of Pdk4 **(A)**, Dhrs9 **(B)**, and Rbp1 **(C)** were quantified by qRT-PCR. Data presented in bar charts are mean ± SEM values from three independent experiments. Groups are significantly different from the control group at **p < 0.01. The groups are significantly different from the IR group at ^#^p < 0.05 and ^##^p < 0.01 determined by Dunnett’s multiple comparisons test.

We also determined the expression of FoxO1 target genes in PA-induced HepG2 cells. As shown in **Figure 8**, the expression of Pdk4 was markedly upregulated in PA-exposed HepG2 cells, when compared with control cells. WEE treatment obviously downregulated the expression of Pdk4 in a concentration-dependent manner. Furthermore, the exposure of HepG2 cells to PA for 24 h significantly decreased the expression of Dhrs9 and Rbp1, and WEE treatment concentration-dependently increased the expression of these two genes in PA-exposed HepG2 cells (see **Figures 8A–C**).

### Effect of WEE on the GLUT2 and GLUT4 Translocation

**Figure 9:**
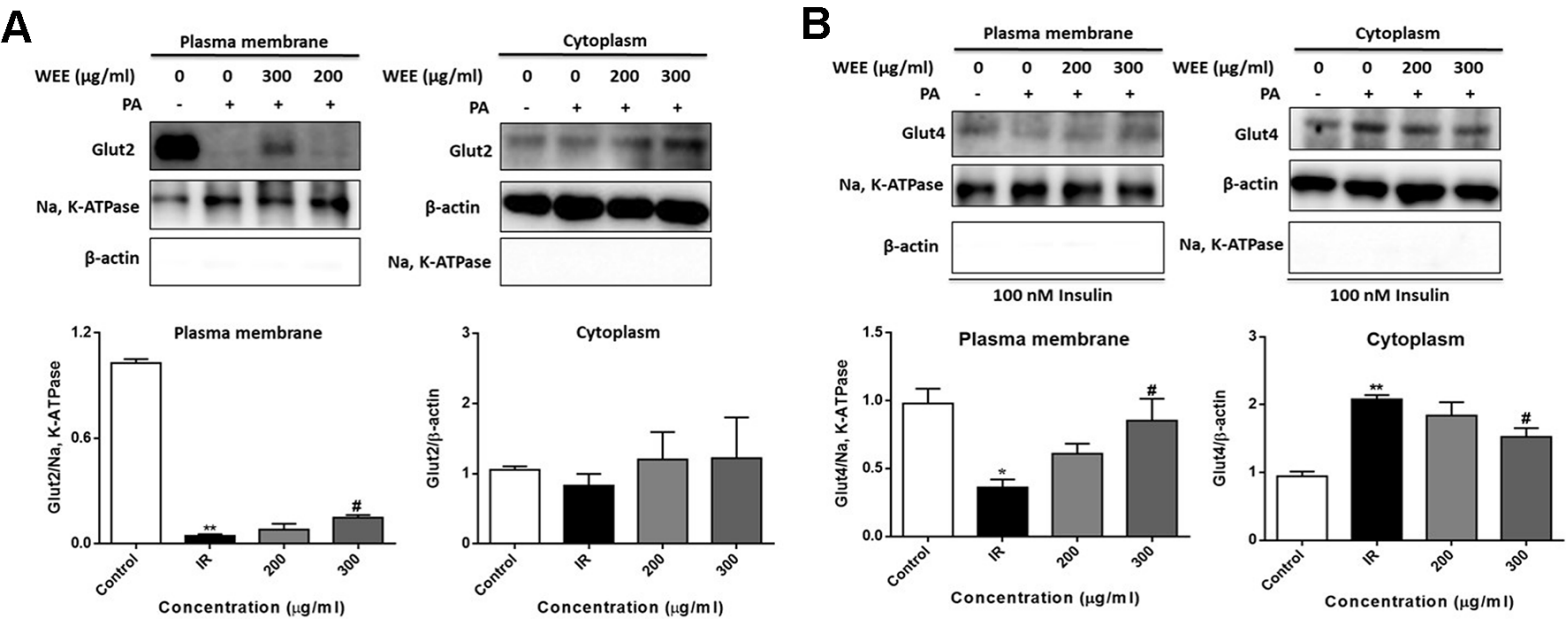
WEE promoted the translocation of GLUT2 and GLUT4 in PA treated HepG2 cells. HepG2 cells were incubated with normal glucose (5.5 mM) or high glucose (30 mM) and PA (0.3 mM) in the absence or presence of WEE (200 and 300 μg/ml) for 24 h. The expression levels of GLUT2 in the plasma membrane and cytoplasm fraction were determined by Western blotting **(A)**. After 24 h treatment and followed by exposure to insulin (100 nM) for 30 min. The expression levels of GLUT4 in plasma membrane and cytoplasm fraction were determined by Western blotting **(B)**. The bar graphs represent the relative expression of GLUT2 and GLUT4 in the plasma membrane and cytoplasm fraction. Data presented in bar charts are mean ± SEM values from six independent experiments. Groups are significantly different from the control group at *p < 0.05, **p < 0.01. The groups are significantly different from the IR group at ^#^p < 0.05 determined by Dunnett’s multiple comparisons test.

GLUT2 is a facilitative glucose transporter located in the plasma membrane of the liver (Wu et al., 1998). To determine whether WEE could induce the GLUT2 translocation, cells were treated with PA and WEE, and the plasma membrane fraction was obtained. The expression level of GLUT2 in the plasma membrane was significantly decreased after PA exposure, and WEE treatment concentration-dependently elevated the GLUT2 expression in the plasma membrane. However, the expression level of GLUT2 was not changed after PA and WEE treatment (see **Figure 9A**).

Insulin stimulates liver glucose uptake through an increase of GLUT4 translocation from intracellular storage vesicles to the plasma membrane, and is a major mechanism for reducing blood glucose level (Huang and Czech, 2007). **Figure 9B** showed that WEE treatment signiﬁcantly increased the GLUT4 expression in the plasma membrane fraction of HepG2 cells. The cytoplasmic protein level of GLUT4 was significantly elevated after PA treatment, and WEE treatment markedly reduced the GLUT4 level in the cytoplasm fraction. We also observed that the expressions of Na, K-ATPase and β-actin in cytosolic and plasma membrane fractions could not be detected, respectively, suggesting that pure cytosolic and plasma membrane fractions were isolated (see **Figures 9A, B**).

## Discussion

In the present study, chlorogenic acid, rutin, and tiliroside were identified in WEE using UPLC analysis. It has been reported that chlorogenic acid has the potential to lower the levels of fasting plasma glucose through the modulation of adiponectin receptor signaling pathways in diabetic mice (Jin et al., 2015). It also ameliorates IR by suppressing autophagy *via* inhibiting the JNK pathway in a fatty liver rat model (Yan et al., 2018). Moreover, rutin can exert anti-IR activity through enhancement of insulin receptor kinase activity and glucose transporter 4 translocation (Hsu et al., 2014). It has been reported that tiliroside can reverse muscle cell IR *via* promoting translocation of glucose transporter 4 (GLUT-4) to the cell surface through its modulation of AMPK/AS160 signaling pathway (Shi et al., 2011; Zhang et al., 2018). Thus, the anti-IR effect of WEE may be, at least in part, due to the presence of these three compounds. Further studies will be conducted to identify other active components responsible for the anti-IR effect of WEE.

Current drug treatment options against T2DM are not satisfactory because of the adverse effects and high cost (Hussein et al., 2004; Salimifar et al., 2013; Harsch et al., 2018). Therefore, researchers are seeking new therapeutic agents with high efficacy and safety. PA, one of the common saturated fatty acids in plasma, has been shown to directly upregulate the inflammatory cytokines and induce lipoapoptosis in hepatocytes (Yan et al., 2014; Ogawa et al., 2018). It has been reported that MTT assay measures metabolic activity as the dye is a substrate for cellular oxidoreductase enzymes (Assad and Jackson, 2019). An increased read-out from the MTT assay after WEE treatment therefore can mean that there are more cells or it can mean there is the same number of cells but they are more metabolically active. Therefore, the total cell number was also calculated and the results were positively correlated with MTT assay. These results suggested that the result of MTT assay indeed reflects the cell viability in our study. Moreover, the results obtained from MTT assay revealed that the cell viability was significantly decreased after exposing HepG2 cells to PA, showing the lipotoxicity medicated apoptosis induced by PA. However, WEE treatment concentration-dependently elevated the cell viability of PA-treated HepG2 cells, suggesting that WEE has a potential to ameliorate PA-induced lipotoxicity. Moreover, IR is reflected by the reduced rates of glucose uptake into the key insulin-sensitive tissues, such as liver and adipose tissue (Honka et al., 2018). Consistent with the previous studies (Yan et al., 2016; Nie et al., 2017), the glucose uptake and consumption in IR cells were decreased when compared to the control cells, indicating that the IR cell model can be successfully built using HepG2 cells. Moreover, the glucose uptake and consumption were markedly elevated after WEE treatment, revealing that WEE has anti-IR potential. In addition, glycogen is the storage form of carbohydrates in mammals, and liver glycogen can be broken down to glucose, which can be transported into blood and maintain the concentration of blood glucose during fasting (Jensen et al., 2011). Some researchers found that the lack of hepatic glycogen increases fat accumulation and promotes the development of liver IR (Irimia et al., 2017). Thus, elevating hepatic glycogen synthesis might be an effective strategy to ameliorate IR. Furthermore, elevated hepatic gluconeogenesis in IR induces excessive glucose production, which plays a pivotal role to hyperglycemia in T2DM (De Souza et al., 2010). Our results showed that the glycogen contents in IR cells were decreased, and WEE treatment increased the glycogen contents in a concentration-dependent manner, suggesting WEE has strong ability to promote the glycogen synthesis in PA-treated HepG2 cells. WEE treatment also inhibited the overproduction of glucose in IR cells, showing that WEE might possess inhibitory effect on gluconeogenesis in PA exposed HepG2 cells. Additionally, increased FFAs in the liver accelerate TG synthesis and induce the intracellular accumulation of FFA-derived lipid intermediates. This will activate the MAPKs, such as JNK, thereby inhibiting the insulin signaling transduction and causing hepatic insulin resistance (Nakamura et al., 2009b). Our present study showed that WEE treatment obviously suppressed the intracellular TG accumulation caused by PA exposure, indicating that WEE may have the ability to ameliorate PA-induced insulin signaling impairment. Considering the above results, we demonstrated that WEE can improve IR caused by PA in HepG2 cells.

The insulin receptor is a member of the ligand-activated receptor/tyrosine kinase family of transmembrane signaling proteins (Lemmon and Schlessinger, 2010). After binding with insulin, insulin receptor undergoes a conformational change and induces activation of the kinase activity in the β subunits (Boucher et al., 2014). This results in phosphorylation of the β subunits, further activating the kinase and allowing phosphorylates the downstream signaling proteins such as the insulin receptor substrate (IRS) proteins, thereby transducing insulin signaling to PI3K/Akt (White, 2003). As the downstream effector of PI3K, the activation of Akt phosphorylates FoxO1, leading to the exclusion of FoxO1 from the nucleus, thus blocking its transcriptional activity and regulating the hepatic gluconeogenic gene expression (e.g., Pdk4, Dhrs9, and Rbp1) (Puigserver et al., 2003; Kane et al., 2011). In our present study, we found that the phosphorylation of IRβ, IRS-1, and Akt were downregulated during PA treatment, and WEE treatment concentration-dependently upregulated the phosphorylated IRβ, IRS-1, and Akt, suggesting that WEE prevented the impairment of insulin-stimulated phosphorylation caused by PA treatment. Moreover, it has been reported that PA-induced ubiquitination of insulin signaling molecules can cause their functional defects and subsequent elicits their proteasomal degradation, thereby leading to the development of IR (Ishii et al., 2015). Consistent with previous study (Ishii et al., 2015), our data showed that the PA treatment induces a significant downregulation of IRβ, IRS-1, and Akt in HepG2 cells. However, WEE treatment inhibited the PA-inducible degradation of these proteins, implying that WEE prevented ubiquitination of the key insulin signaling molecules in PA-exposed HepG2 cells. Moreover, we also found that the nuclear translocation and phosphorylation of FoxO1 were also inhibited by the WEE treatment in PA-treated HepG2 cells, revealing that WEE treatment prevented Akt/FoxO1 signaling. Notably, PA-induced localization of FoxO1 into the nucleus resulted in the alteration of the transcription activities of its target genes, including Pdk4, Dhrs9, and Rbp1; WEE treatment successfully reversed PA-induced changes of these genes, suggesting that the inhibitory effect of WEE on gluconeogenesis in hepatocytes may be related to the suppression of FoxO1 signaling, thereby regulating the gluconeogenic genes expression.

Moreover, MAPKs (consisting of JNK, ERK, and p38) are also responsible for the feedback phosphorylation of IRS-1 tyrosine and inhibit the signal transduction of insulin receptor (Hers and Tavare, 2005; Gao et al., 2010; Bi et al., 2013). In line with the previous study (Hers and Tavare, 2005; Gao et al., 2010; Bi et al., 2013), after treatment with PA for 24 h, the phosphorylation of JNK, ERK, and p38 was remarkably elevated in HepG2 cells. WEE treatment inhibited the phosphorylated JNK, ERK, and p38 in a concentration-dependent manner. These results suggested that WEE possesses strong suppressive effect on MAPKs signaling, thereby promoting the IRS-1 signaling transduction.

Furthermore, Akt inhibits the activation of GSK-3β through phosphorylation at Ser9 residue (Cross et al, 1995). GSK-3β is an enzyme characterized by its ability to phosphorylate and impair glycogen synthesis in hepatocytes (Rui, 2014). Some studies showed that GSK-3β has potential pro-apoptotic action through regulating an array of transcription factors (e.g., c-Jun and CREB) and downregulating the expression of cell survival genes (Boyle et al., 1991; Nikolakaki et al., 1993; Fiol et al., 1994; Jensen et al., 2006). In our present study, we observed that the phosphorylation of GSK-3β at ser9 was remarkably elevated by WEE treatment in PA-exposed HepG2 cells, indicating that WEE effectively inhibited the activation of GSK3β, thus promoting glycogen synthesis. We also found that the phosphorylated c-Jun and CREB were significantly upregulated in PA-exposed HepG2 cells, and WEE treatment concentration-dependently downregulated the phosphorylated c-Jun and CREB, revealing that the anti-lipotoxicity ability of WEE may be related to the inhibitory effect on GSK3β/CREB/c-Jun signaling.

GLUT2 is the major hepatic glucose transporter and is a low affinity, high capacity transporter expressed in high levels on the sinusoidal membranes of hepatocytes (Thorens, 1996). It has been reported that insulin receptor and GLUT2 form a receptor-transporter complex in the plasma membrane of hepatocytes (Eisenberg et al., 2005), and such insulin receptor GLUT2 complexes were increased to improve hepatic insulin sensitivity (Gonzalez-Rodriguez et al., 2008). Consistent with previous study, we found that the expression of GLUT2 in the plasma membrane was significantly decreased after PA treatment (Jayanthy and Subramanian, 2015), and WEE treatment concentration-dependently elevated the expression of GLUT2 in the plasma membrane, indicating that WEE treatment enhances the glucose uptake mainly *via* promoting GLUT2 translocation in PA treated HepG2 cells.

GLUT4 is considered as the one of the most important type of glucose transporter in mediating insulin-dependent glucose uptake and maintaining glucose homeostasis (Wallberg Henriksson and Zierath, 2001). The activation of Akt induced by insulin accelerates the translocation of GLUT4 vesicles to the plasma membrane, which results in enhanced glucose uptake in the adipose and muscle cells (Gonzalez and McGraw, 2006). Notably, it has been reported that GLUT4 translocation into plasma membrane promotes hepatic glucose uptake, and insulin treatment promoted the translocation of GLUT4 in HepG2 cells (Wang et al., 2014). Our results showed that GLUT4 migration from cytosol to membrane induced by insulin was prevented by PA treatment. WEE treatment markedly elevated the GLUT4 expression in the plasma fraction of HepG2 cells, revealing that WEE may partly elevate the glucose uptake in PA exposed HepG2 cells by increasing GLUT4 translocation.

There are two limitations of our study that should be mentioned. Firstly, lack of *in vivo* data represents a major limitation of the study for the investigation of the anti-IR action of WEE. Future investigations will be designed to perform high fat diet induced IR animal model to validate *in vitro* data. Secondly, in this study, we only investigated the anti-IR effect of WEE in PA treated hepatocytes. However, other insulin sensitive cells, such as muscle cells and adipocytes, are not tested, which could not comprehensively evaluate the anti-IR effect of WEE treatment on all target cells. Therefore, further studies are required to demonstrated that the anti-IR ability in muscle cells and adipocytes.

## Conclusion

**Figure 10:**
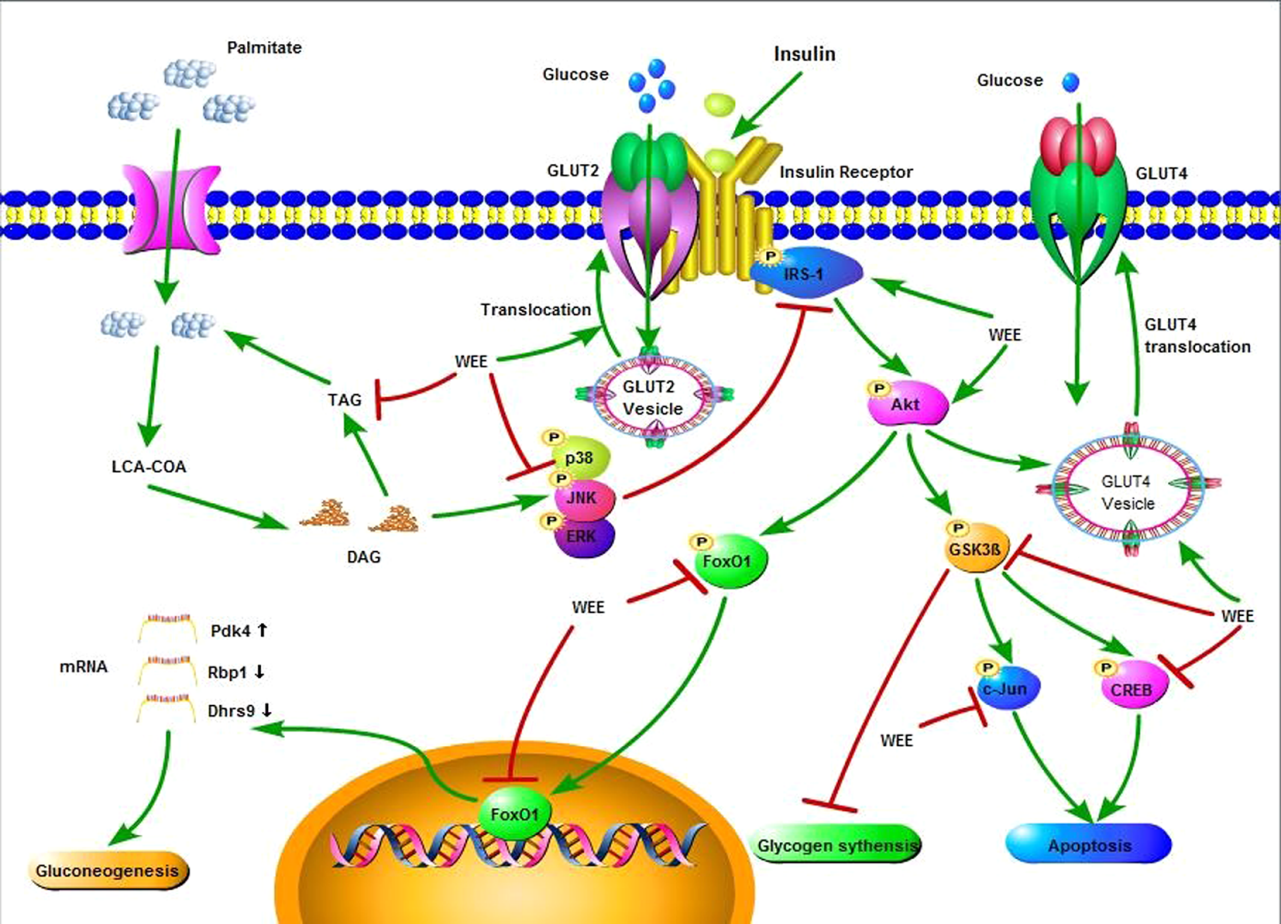
Insulin signaling cascade targeted by WEE in PA treated HepG2 cells.

In summary, the current study showed that WEE possesses inhibitory effect on PA-induced lipotoxicity in HepG2 cells. Moreover, WEE treatment markedly elevated the glucose consumption and uptake in HepG2 cells treated with PA. The endogenous glucose production and glycogen content were decreased and increased after WEE treatment in PA-treated HepG2 cells, respectively. The intracellular TG content was also suppressed by WEE treatment. Mechanistic investigation showed that the regulation of IRS-1/GSK3β/FoxO1 signaling pathway associated with the anti-IR effect of WEE in PA-exposed HepG2 cells (**Figure 10**), and the translocation of GLUT2 and GLUT4 was promoted by WEE treatment. The results provide further pharmacological basis for the clinical application of WEE in the treatment of IR-associated metabolic diseases.

## Data Availability Statement

All datasets generated for this study are included in the article/supplementary material.

## Author Contributions

Design of the study: TL, SZ. Conduct of all the experimental studies: YZ, LY, YD. Data collection: YZ, GL. Data analysis: YZ, LY. Data interpretation: YZ, LY: Manuscript writing: YZ, BC. Manuscript revising: BC, TL, and SZ. All authors read and approved the final manuscript.

## Conflict of Interest

Author BC was employed by the Quality Healthcare Medical Services.

The remaining authors declare that the research was conducted in the absence of any commercial or financial relationships that could be construed as a potential conflict of interest.
